# Anticancer Effect of Metformin in Herceptin-Conjugated Liposome for Breast Cancer

**DOI:** 10.3390/pharmaceutics12010011

**Published:** 2019-12-20

**Authors:** Ji-Yeon Lee, Dae Hwan Shin, Jin-Seok Kim

**Affiliations:** 1 Drug Information Research Institute (DIRI), College of Pharmacy, Sookmyung Women’s University, Cheongparo 47 gil 100, Yongsan gu, Seoul 04310, Korea; jiyeonlee0325@gmail.com; 2 College of Pharmacy, Chungbuk National University, Cheongju 28160, Korea; dshin@chungbuk.ac.kr

**Keywords:** Metformin, Herceptin, breast cancer stem cell, liposome, targeting

## Abstract

Metformin (MET) is an anti-diabetic drug effective against breast cancer, targeting breast cancer stem cells (BCSCs). MET-encapsulating liposome (LP-MET) and Herceptin-conjugated LP-MET (Her-LP-MET) were evaluated for their anti-cancer effect in vitro and in vivo. Size and zeta potentials of LP-MET and Her-LP-MET were suitable for enhanced permeability and retention effects. Her-LP-MET yielded greater inhibition of BCSC proliferation in vitro than free MET or LP-MET, as well as a dose-dependent long-term anti-proliferation effect. Further, the anti-migration effect of Her-LP-MET on BCSCs was superior to that of MET or LP-MET, and was enhanced when used in concert with doxorubicin (DOX). In a mouse model, Her-LP-MET combined with free DOX was more effective than free MET, free DOX, or Her-LP-MET. Moreover, Her-LP-MET combined with free DOX yielded tumor remission, whereas free DOX alone resulted in metastasis or death. As such, Her-LP-MET formulation is expected to provide a new therapeutic modality targeting BCSCs.

## 1. Introduction

Breast cancer is a common type of female cancer, constituting the second leading cause of death for women. Recent studies have shown that breast cancer stem cells (BCSCs) are a main indicator of poor breast cancer prognosis, since unlike cancer cells, cancer stem cells (CSCs) are closely related to cancer initiation and metastasis [1,2,3,4]. CSCs can regenerate various cancer cell types during radiation therapy and chemotherapy, resulting in relapse [5,6]. Therefore, drugs that selectively target the CSCs are thought to play a crucial role in determining long-term survival of cancer patients [7,8].

Metformin (MET or 1,1-dimethylbiguanide hydrochloride) is traditionally used as an anti-type 2 diabetic agent which stimulates AMP-activated protein kinase (AMPK), leading to the inhibition of the mTOR pathway [9,10]. Interestingly, diabetes patients treated with MET have been shown to have a reduced cancer risk, although it is unclear whether MET affects cancer directly or indirectly [11,12,13,14,15]. MET has become an object of research focus due to its ability to target CSCs, which affect chemoresistance, cell proliferation, self-renewal, differentiation, metastasis, metabolism, and tumor relapse [16]. Recent studies have demonstrated that low-dose MET can affect breast cancer by specifically targeting BCSCs [17,18,19,20] However, MET is limited by low bioavailability due to its short half-life, high hydrophilicity, and non-selective biodistribution [21]. As such, an improved understanding of MET’s anti-cancer mechanism will help to optimize its treatment conditions as a monotherapy or in combination with other cancer treatment strategies.

Liposomes are core-shell artificial phospholipids that can deliver both hydrophilic and lipophilic drugs simultaneously as well as accomplish drug targeting through the conjugation of specific ligands to their surfaces [22,23]. The main advantage of applying liposomes is to protect the drugs from degradation. This contributes to increase the circulation time of the drugs and the likelihood of achieving partial or full selectivity. We adopted liposome for MET and liposomal formulation of MET for injection is expected to overcome the poor oral bioavailability of the drug owing to its short in vivo half-life, hydrophilic property, and non-selective drug distribution [21,24].

Human epidermal growth factor receptor 2 (HER2) is a transmembrane receptor tyrosine kinase that controls critical cellular functions such as differentiation, cell growth, and survival in normal and malignant breast epithelial cells. About 20–30% of breast cancer patients show overexpression of the HER2 gene, which is associated with aggressive clinical phenotype, increased recurrence, and unfavorable prognosis. Since CSC also overexpresses HER2 even in breast cancers not categorized as HER2-positive, HER2-directed treatments using Herceptin^®^ (trastuzumab) are indicated for numerous patients with both HER2-positive and HER2-negative breast cancer [25].

Recently, Korkaya et al. reported that HER2 is an important regulator of the CSC population in breast cancer, where HER2 overexpression increases CSC population while HER2 blockade decreases it both in vitro and in vivo [25,26]. In addition, in human breast cancers, there is a correlation between HER2 amplification and CSC population indicated by expression of the BCSC marker ALDH-1 [27]. Furthermore, preferential killing of CD44^+^/CD24^−/low^ breast cancer cells or BCSCs induced by MET can be sufficient to overcome the primary resistance to Herceptin^®^ in HER2+ breast cancer xenografts. Another study has also demonstrated a synergistic anti-cancer effect of MET with Herceptin [18,28].

We hypothesized that Herceptin-conjugated immunoliposome encapsulating MET can target both HER2+ breast cancer cells and BCSCs via active targeting of a well-known specific receptor and endocytic activity of CSCs, respectively. We also investigated the direct inhibition of growth of breast cancer cells after binding of Herceptin and differentiation of BCSCs into breast cancer cells by MET, followed by the killing of these cells with doxorubicin (DOX).

## 2. Materials and Methods

### 2.1. Materials and Reagents

Metformin HCl, doxorubicin HCl, cholesterol, accutase, poly-2-hydroxyethyl methacrylate (polyHEMA), 3-(4,5-dimethylthiazol-2-yl)-2,5-diphenyltetrazolium bromide (MTT), crystal violet, dimethyl sulfoxide (DMSO), bovine serum albumin (BSA), glutaraldehyde, insulin solution, human epidermal growth factor (hEGF), basic fibroblast growth factor (bFGF), methanol, and chloroform were purchased from Sigma-Aldrich Co. (St. Louis, MO, USA). Egg L-α-phosphatidylcholine (EPC), 1,2-distearoyl-*sn*-glycero-3-phosphoethanolamine-N-[maleimide(polyethyleneglycol)-2000] (ammonium salt) (mal-PEG-DSPE), and 1,2-distearoyl-*sn*-glycero-3-phosphoethanolamine-N-[methoxy(polyethylene glycol)-2000] (ammonium salt) (mPEG-DSPE) were purchased from Avanti Polar Lipids, Inc. (Alabaster, AL, USA). Herceptin was purchased from Roche (Basel, Switzerland). Dulbecco’s modified eagle medium (DMEM), penicillin/streptomycin, trypsin-EDTA, Dulbecco’s phosphate buffered saline (DPBS), and fetal bovine serum (FBS) were purchased from WelGENE Inc. (Gyeongsan, Korea). 17β-estradiol pellets were purchased from Innovative Research of America (Sarasota, FL, USA). B-27 supplement and DMEM/F12 were purchased from CureBio Co., Ltd. (Seoul, Korea). A bicinchoninic acid (BCA) protein assay kit was purchased from BIO-Rad Laboratories, Inc. (Hercules, CA, USA). Anti-CD24-PE antibody and anti-CD44-FITC antibody were purchased from Miltenyi Biotec (Bergisch Gladbach, Germany). All reagents used were analytical or clinical grade.

### 2.2. Methods

#### 2.2.1. Preparation of Herceptin Conjugated-Pegylated Liposome Incorporating MET (Her-LP-MET) by Post-Insertion Method

To prepare PEGylated liposome incorporating MET (LP-MET), a liposome was prepared using thin lipid film evaporation technique followed by freeze-thaw protocols. The liposome was composed of EPC, mPEG-DSPE, and cholesterol at 6:0.5:3.5 molar ratio. The lipid mixture (10 mM) was dissolved in chloroform and evaporated gently to make a lipid film under nitrogen gas flow using a rotary evaporator (Laborota 4000; Heidolph, Schwabach, Germany) and the dried film was hydrated with HEPES buffer (20 mM, pH 7.5) containing 10 mM MET HCl. The encapsulation efficiencies of resulting suspension were enhanced using ten freeze-thaw cycles [29], followed by extrusion through a cellulose membrane filter (Whatman; Pittsburgh, PA, USA). The unincorporated MET was removed by centrifugation.

To prepare Herceptin-conjugated liposome (Her-LP), EPC, mal-mPEG-DSPE, and cholesterol at 6:0.5:3.5 molar ratio was dissolved in chloroform and made into a lipid film by removal of chloroform in an evaporator. The dried lipid film was hydrated in HEPES buffer and vortexed. The liposome was sonicated and the resulting suspension was extruded. Herceptin (1000 μg/mL) was thiolated by reacting with 1 mg/mL Traut’s reagent, at a molar ratio of 10:1 Traut’s reagent to Herceptin. Thiolated Herceptin was purified and then mixed with prepared liposome corresponding to a 1:10 molar ratio of Herceptin:liposome.

**Figure 1:**
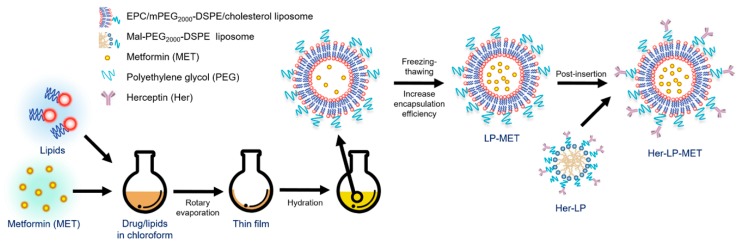
Preparation of Herceptin-conjugated LP-MET (Her-LP-MET).

Finally, Her-LP was incubated with prepared LP-MET at a molar ratio of 0.02:1 for 2 h at 60 °C. The mixture was cooled and purified by centrifugation to obtain Her-LP-MET (Figure 1).

#### 2.2.2. Size Distribution and Zeta Potential Analysis

Size distribution and zeta potential of liposomal formulations were measured using NICOMP 380ZLS dynamic laser light scattering (DLS) system (Santa Barbara, CA, USA). The formulation sample was diluted in water to 1:100 volume ratio and analyzed at ambient temperature with a scattering angle of 90°.

#### 2.2.3. Encapsulation Efficiency

The encapsulation efficiency of MET into liposome was measured as per Bligh and Dyer extraction method [30]. Briefly, 100 μL liposome suspension was vortexed with 150 μL DPBS, 250 μL methanol, and 1 mL chloroform, then centrifuged at 2700 *g* for 15 min to achieve two-phase separation of hydrophilic materials, including MET and lipophilic materials. The lipophilic phase was removed using a syringe, then 1 mL fresh chloroform was mixed with hydrophilic phase and the mixture was centrifuged again. This process was repeated three times, after which the amount of MET in hydrophilic phase was determined using a spectrophotometer at 230 nm. The encapsulation efficiency of MET (%) in liposome was calculated using the following equation:

$$\mathrm{Encapsulation}\;\mathrm{efficiency}\;\mathrm{of}\;\mathrm{MET}\;\left(\%\right)=\left(\frac{C_{i}-C_{\mathrm{un}}}{C_{i}}\right)\times 100\%$$

where *C*_i_ represents the initial concentration of MET from liposome and *C*_un_ represents the concentration of unencapsulated MET.

#### 2.2.4. Culture of MCF-7 Human Breast Cancer Cells

MCF-7 cells were purchased from Korean Cell Line Bank (KCLB, Seoul, Korea). They were maintained in DMEM supplemented with 10% FBS and 100 unit/mL penicillin/streptomycin. The cells were grown at 37 °C in an incubator (5% CO_2_, Sanyo Electric Co. Ltd., Osaka, Japan) to approximately 80% confluence, then washed with DPBS, trypsinized, and collected. The collected cells were centrifuged and resuspended with fresh DMEM containing 10% FBS in culture plates. The medium was replaced periodically.

#### 2.2.5. Culture of Breast Cancer Stem Cells (BCSCs)

For culture of BCSCs, the cell culture plates were coated with polyHEMA and the medium was prepared with DMEM-F12 containing 5 μg/mL insulin, 0.4% BSA, 20 ng/mL hEGF, 20 ng/mL bFGF, and B-27 supplement. In the medium for BCSCs, MCF-7 cells grew as nonadherent spheres, labeled mammospheres, and increased the fraction of BCSCs. Fresh culture medium was added every 2 days. BCSCs were harvested on day 7, centrifuged, and dissociated to single cells using accutase for experiments.

#### 2.2.6. Flow Cytometric Analysis of Breast Cancer Stem Cells (BCSCs)

BCSCs and MCF-7 cells were washed with DPBS three times and dissociated into single cells. The single MCF-7 cells and BCSCs were mixed with 5 μL anti-CD24-PE antibodies and 5 μL anti-CD44-FITC antibodies in DPBS for 30 min at 4 °C. Then, stained cells were rinsed and resuspended in 0.5 mL cold DPBS. Flow cytometric detection of CD44^+^/CD24^−/low^ markers were performed using FACSCalibur™ (BD Bioscience, San Jose, CA, USA).

#### 2.2.7. Regulation of CD44^+^/CD24^−/low^ Cells by Metformin (MET)

Differentiation of the BCSCs by MET was analyzed using flow cytometry. The dissociated BCSCs were seeded in polyHEMA-coated 6-well plates at 10,000 cells/well density. The cells were incubated with 7 mM MET medium for 0, 24, and 72 h. Then, single cells dissociated with accutase were rinsed with DPBS. Single BCSCs were mixed with 5 μL anti-CD24-PE and 5 μL anti-CD44-FITC in DPBS for 30 min at 4 °C. The stained cells were then rinsed three times and resuspended in 0.5 mL cold DPBS for flow cytometry.

#### 2.2.8. Anti-Proliferation in MCF-7 Cells and Breast Cancer Stem Cells (BCSCs)

Inhibition of cellular proliferation by MET was determined using MTT assay. The dissociated MCF-7 cells and BCSCs were added in 96-well plate at 3000 cells/100 μL serum-containing medium density in each well. After overnight incubation, the medium was aspirated and the cells in each well were treated with DMSO, HEPES, 10 mM MET, 10 mM LP-MET, Her-LP, and 10 mM Her-LP-MET in medium. After incubation for 24 and 48 h, each well was incubated with MTT solution for 4 h. Then, the supernatant was aspirated and 100 μL DMSO was added. Plates were incubated at 37 °C for 5 min and gently agitated using FINE CR 100 shaking plate (Finemould Precision IND. CO., Seoul, Korea) for 10 min to dissolve the formed formazan crystal. Absorbance at 570 nm was measured using a 1420 Multilabel Counter (Victor3; PerkinElmer, Waltham, MA, USA). Cell growth (% of control) was calculated using the following equation:

$$\mathrm{Cell}\;\mathrm{growth}\;\left(\%\;\mathrm{of}\;\mathrm{control}\right)=\frac{{\mathrm{OD}}_{570\left(\mathrm{Sample}\right)}-{\mathrm{OD}}_{570\left(\mathrm{Original}\right)}}{{\mathrm{OD}}_{570\left(\mathrm{Control}\right)\;}-{\;\mathrm{OD}}_{570\left(\mathrm{Original}\right)}}\times 100$$

where OD_570(Sample)_ represents the absorbance of the wells treated with the various formulations of MET, OD_570(Original)_ represents the absorbance of the wells at the time of drug formulation treatment, and OD_570(Control)_ represents the absorbance of the wells treated with HEPES.

#### 2.2.9. Clonogenic Assay

A clonogenic assay was carried out to evaluate the colony-forming ability [31]. BCSCs (2000 cells/well) were seeded in 6-well plates. After incubation for 4 h, HEPES, 10 mM MET, 1 μM DOX, 10 mM LP-MET, 10 mM Her-LP-MET, and Her-LP-MET+DOX were treated to each well. For Her-LP-MET+DOX, 10 mM Her-LP-MET, and 1 μM DOX were treated individually. After 2 weeks, the medium was removed and cells were fixed with 6.0% (*v*/*v*) glutaraldehyde. Crystal violet solution (0.5%, *w*/*v*) was treated to each well for 30 min. Then colonies were rinsed with clean water and number of colonies was counted using a microscope. The colony formation (% of control) was calculated according to below equation.

$$\mathrm{Colony}\;\mathrm{formation}\;\left(\%\;\mathrm{of}\;\mathrm{control}\right)=\frac{\mathrm{number}\;\mathrm{of}\;\mathrm{colonies}\;\mathrm{after}\;\mathrm{treatment}}{\mathrm{number}\;\mathrm{of}\;\mathrm{colonies}\;\mathrm{in}\;\mathrm{control}\;\left(\mathrm{DPBS}\right)}\times 100$$

#### 2.2.10. Anti-Migration Assay

Cell migration was evaluated according to wound-healing assay. BCSCs were cultured in 24-well plates until confluent, then wounds were made by scratching linearly with a SPLScar™ Scratcher on confluent cells. Each well was washed with DPBS three times to remove detached cells. The wounded cells were treated with HEPES, 10 mM MET, 1 μM DOX, 10 mM LP-MET, 10 mM Her-LP-MET, and Her-LP-MET+DOX. For Her-LP-MET+DOX, 10 mM Her-LP-MET and 1 μM DOX were treated individually. After incubation for 24 h, cells were washed and observed using a microscope. Percent migration was calculated using the equation:

$$\mathrm{Migration}\;\left(\%\;\mathrm{of}\;\mathrm{control}\right)=\frac{\mathrm{initial}\;\mathrm{width}-\mathrm{final}\;\mathrm{width}}{\mathrm{initial}\;\mathrm{width}}\times 100$$

where initial width indicates the empty area (in wounded cells) at the moment of sample treatment and final width gives the empty area (in migrated cells) after 24 h.

#### 2.2.11. In Vivo Anti-Cancer Studies

Animal experiments were performed using 6-week-old female BALB/c-nu mice (25–30 g) which were purchased from Saeronbio Inc. (Uiwang, Korea). The animals were maintained in an animal facility at 23 °C, 50% relative humidity with appropriate light/dark cycle with free access to water and food. BCSCs (2 × 10^6^) were administrated subcutaneously into the right flank of mice, and a 17β-estradiol pellet (0.18 mg/pellet) was implanted subcutaneously using a trocar to establish a breast cancer disease model. All the mice developed tumors of approximately 60 mm^3^ within 10 days. The mice were randomly distributed into five groups (*n* = 5 per group), as an untreated control group and experimental groups receiving MET at 10 mg/kg, DOX at 4 mg/kg, LP-MET at 10 mg/kg, Her-LP-MET at 10 mg/kg, or Her-LP-MET at 10 mg/kg + DOX at 4 mg/kg. Treatments were applied via intravenous injection every 3 days (three cycles). The size of tumor was measured using Vernier caliper at 2–3-day intervals and calculated using the below standard formula.

$$\mathrm{Tumor}\;\mathrm{size}=\frac{{\left(\mathrm{width}\right)}^{2}\times \mathrm{length}\;}{2}$$

All mouse experiments were carried out according to Animal Care and Use Committee procedures and guidelines of Sookmyung Women’s University, Korea. (Identification number: SMWU-IACUC-1510-020, Date: 22 Oct 2015)

#### 2.2.12. Statistical Analysis

All results are presented as mean value ± standard deviation (SD). Statistical analysis was performed using a Student’s *t* test. Statistical significance was determined for *p* < 0.05 (95% confidence interval) or *p* < 0.01 (99% confidence interval).

## 3. Results

### 3.1. Size Distribution and Zeta Potential Analysis

Mean tumor diameters were 52 ± 32, 132 ± 49, and 159 ± 17 nm for Her-LP, LP-MET, and Her-LP-MET-treated mice, respectively (Table 1). This size, ranging from 100 to 200 nm, is appropriate for improving the biodistribution of liposomes to tumor tissues (EPR effect) [32]. Zeta potentials were—−4.8 ± 4.2 mV, −8.7 ± 1.3 mV, and −10.6 ± 7.6 mV for Her-LP, LP-MET, and Her-LP-MET, respectively.

### 3.2. Encapsulation Efficiency (%) of Metformin (MET)

The encapsulation efficiency of MET was calculated by determining the absorbance at 230 nm. Encapsulation efficiency of MET into Her-LP-MET was 21.6 ± 2%.

### 3.3. Cell Culture of MCF-7 Cells and Breast Cancer Stem Cells (BCSCs)

**Figure 2:**
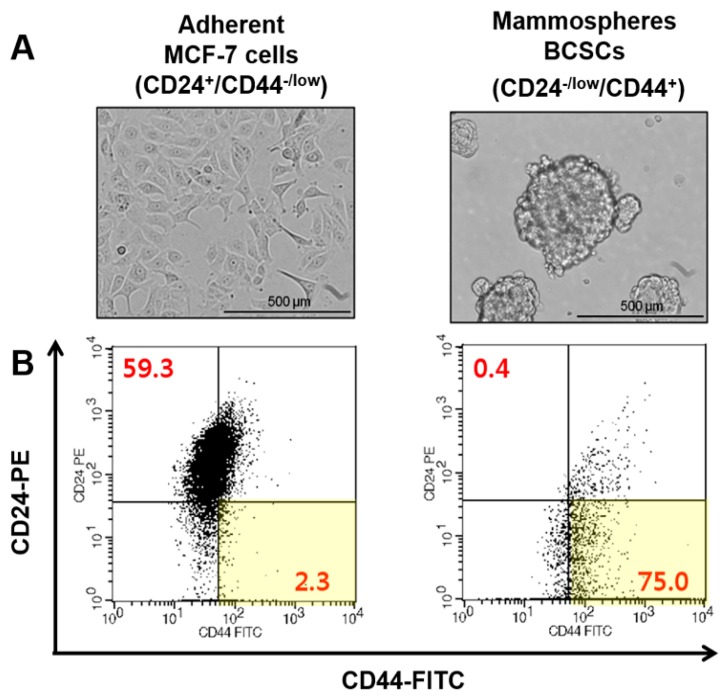
(**A**) Images of MCF-7 cells and BCSCs under a light microscope; (**B**) flow cytometric characterization of phenotypes for MCF-7 cells and BCSCs using anti-CD44-FITC and anti-CD24-PE staining.

MCF-7 cells adhered to the bottom of culture plate when they grew, whereas BCSCs grew as nonadherent mammospheres in poly-HEMA coated culture dish (Figure 2A). The population of CD44^+^/CD24^−/low^ cells was identified as BCSCs. The percentage of BCSCs from mammospheres was 75.0%, and 2.3% of MCF-7 cells (Figure 2B).

### 3.4. Regulation of CD44^+^/CD24^−/low^ Cells by Metformin (MET)

**Figure 3:**
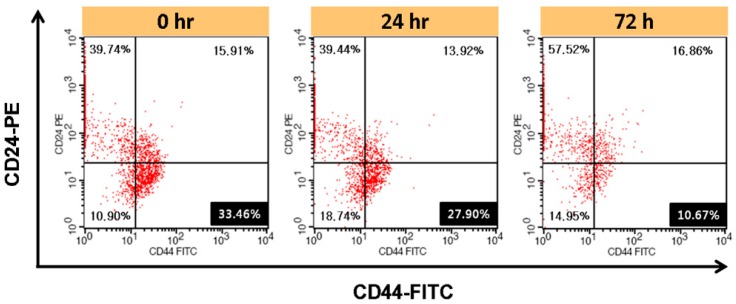
Regulation of CD44^+^/CD24^−/low^ cells by MET. Cells were treated with 7 mM MET for 24 and 72 h and stained with anti-CD44-FITC and anti-CD24-PE prior to flow cytometric analysis.

After incubation for 72 h, the population of BCSCs (CD44^+^/CD24^−/low^) about 3.1 times lower (33.46% to 10.67%) under MET treatment compared to untreated cell culture (Figure 3).

### 3.5. Anti-Proliferation in MCF-7 Cells and Bcscs

**Figure 4:**
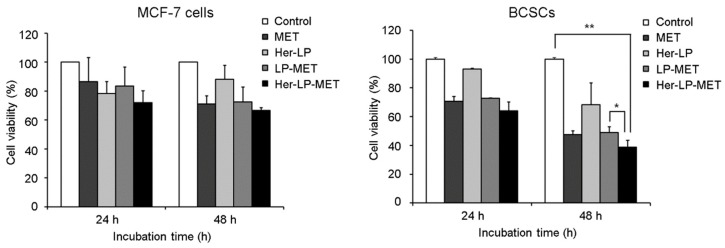
Inhibition of proliferation determined by MTT assays of MCF-7 cells (left panel) and breast cancer stem cells (BCSCs) (right panel). Both cells were incubated with control, MET, Her-LP, LP-MET, or Her-LP-MET, or used as controls, for 24 and 48 h. Data were calculated as a percentage of the control. Single asterisk (*) indicates *p* < 0.05; double asterisks (**) indicate *p* < 0.01.

MET inhibited growth of BCSCs much more effectively than MCF-7 cells (Figure 4). There was no significant difference between MET and LP-MET (71.1% vs. 72.6%) in MCF-7 cell growth at 48 h, indicating that the liposomes caused no significant cytotoxicity. Growth was decreased with Her-LP-MET (66.5%). In BCSCs, Her-LP-MET showed the highest growth inhibition (61.3%) among the treated BCSC groups. Meanwhile, growth inhibition of BCSCs by MET and LP-MET was 52.5% and 51.1%, respectively. This result suggests that addition of Herceptin enhanced the growth inhibition of LP-MET on BCSCs.

### 3.6. Clonogenic Assay

**Figure 5:**
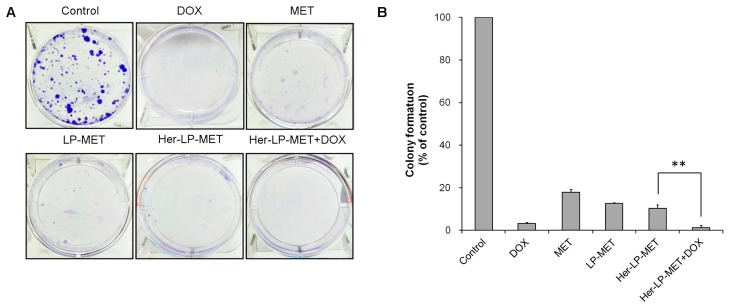
Clonogenic assay for BCSCs treated with various doxorubicin (DOX) and Metformin (MET) formulation for 2 weeks (**A**). Colony formation was calculated as percentage of control (**B**). Asterisks ** indicate *p* < 0.01.

Clonogenic assay of BCSCs’ colonial development indicated that Her-LP-MET treatment yielded considerably smaller colonies over a two-week period of incubation, showing colony numbers 10.4% of the control group. Her-LP-MET+DOX further suppressed colony formation, showing colony numbers only 1.2% of the control group, and showed the most effective inhibition of colony formation among the tested treatments (Figure 5).

### 3.7. Anti-Migration Assay

**Figure 6:**
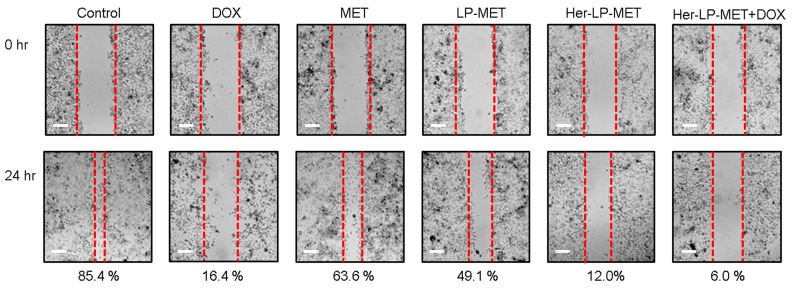
Migration assay of breast cancer stem cells (BCSCs) treated with control and various MET and DOX formulations for 24 h. The rate of migration was calculated as percentage of initial width at 0 h. (white scale bar: 200 μm, 4× magnification)

The wound-healing of cells treated with Her-LP-MET+DOX was strongly inhibited, showing 6.0% migration compared to control, followed by Her-LP-MET (12.3%) and DOX (16.4%) (Figure 6).

### 3.8. In Vivo Anti-Cancer Studies

**Figure 7:**
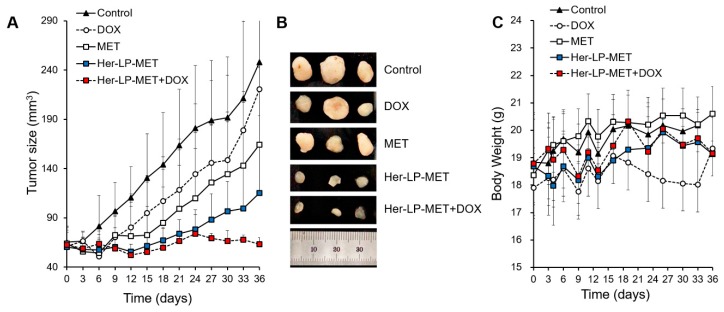
(**A**) Change of mean tumor size in control, MET, DOX, Her-LP-MET, and Her-LP-MET+DOX-treated mice; (**B**) Representative tumor photographs that developed in the xemograft model of BCSCs untreated or treated with various MET and DOX formulations; (**C**) changes in body weight in control, MET, DOX, Her-LP-MET, and Her-LP-MET+DOX-treated mice.

Tumor size and mean body weight of mouse after intravenous administration of various MET formulations into xenograft nude mice are described in Figure 7. Her-LP-MET+DOX showed the strongest anti-cancer effect, followed by Her-LP-MET and DOX. This is presumably because Herceptin-attached liposomes increased the circulation of MET in the blood, enhancing its anticancer ability. This combined therapy reduced the tumor mass and prolonged tumor remission much more effectively than drugs alone in a xenograft mouse model.

## 4. Discussion

Although overall mortality in breast cancer patients has decreased, breast cancer is the second most common female cancer [33]. One therapeutic strategy for breast cancer therapy is targeting the BCSCs, which are regarded to play an important role in both cancer development and metastasis. As such, drugs that selectively target CSCs can promote long-term survival of cancer patients.

MET has been known to control the growth of breast cancer cells in low dose by targeting BCSCs specifically [19]. However, clinical application of MET has been limited due to its low bioavailability caused by high hydrophilicity, short half-life, and non-selective biodistribution [21]. We developed a Herceptin-conjugated PEGylated liposome incorporating MET (Her-LP-MET) for efficient drug delivery and specific targeting to the BCSCs. We also investigated the anticancer efficiency of combined treatment with Her-LP-MET and DOX in vitro and in vivo.

Physicochemical properties of LP-MET, Her-LP, and Her-LP-MET were characterized in terms of particle diameter size, zeta potential, and encapsulation efficiency. The mean diameters of LP-MET, Her-LP, and Her-LP-MET were 132 ± 49 nm, 52 ± 32 nm, and 159 ± 17 nm, respectively (Table 1). The slightly larger particle size of Her-LP-MET compared to LP-MET was presumably due to the apparent size increase due to the addition of Herceptin on the surface of the liposome. Particle size is closely related to the enhanced permeability and retention (EPR) effect, with this effect optimized at 100–200 nm [34]. The measured zeta potential of LP-MET, Her-LP, and Her-LP-MET were in the range of 0 to −10 mV. Nanoparticles with mild negative charge are less toxic after intravenous administration compared to positively charged particles [34,35], suggesting that both the particle size and zeta potential of Her-LP-MET are ideal for intravenous administration. In addition, the use of freeze–thaw cycles enhanced the encapsulation efficiency of Her-LP-MET, in spite of MET’s hydrophilicity.

Breast cancer cells with CD44^+^/CD24^−/low^ specifically have CSC properties [3,36,37,38]. Prior to investigating the delivery of Her-LP-MET to BCSCs, the population of BCSCs was also identified using FACS analysis by determining portion of CD44^+^/CD24^−/low^. The percentage of CD44^+^/CD24^−/low^ portion in BCSC mammospheres was 75.0%, 32-fold higher than that of MCF-7 cells at 2.3% (Figure 2). This is consistent with previous studies showing that cultured mammospheres had a high proportion of CD44^+^/CD24^−/low^ BCSCs [3,37,38,39].

Regulation of BCSCs by MET was analyzed using flow cytometric analysis. After 72 h treatment with 7 mM MET, the CD44^+^/CD24^−/low^ portion decreased to a third of the control, indicating that MET dramatically reduced the BCSC portion of mammosphere cells (Figure 3). This result indicates that MET treatment can regulate CSCs and lead to cell death, as consistent with the results in previous studies [18].

The short-term anti-cancer activity of control and various MET and DOX formulations was determined using in vitro MTT assay with MCF-7 cells and BCSCs for 24 and 48 h. Proliferation of BCSCs was suppressed more effectively by MET compared to MCF-7 cells (Figure 4). We hypothesize that MET preferentially kills breast cancer initiating CD44^+^/CD24^–/low^ cell subpopulation, leading to increased cell death. In MCF-7 and BCSCs at 24 and 48 h, there were no significant differences in proliferation between MET and LP-MET treatment, showing that simple encapsulation of MET into liposomes did not influence its in vitro anticancer efficacy for either cell type. Since the liposome was composed of non-toxic lipids such as EPC, mPEG-DSPE, and cholesterol, and the zeta potential of prepared liposome was mild negative, liposome as a vehicle itself exerts no cytotoxicity. Finally, treatment with Her-LP-MET showed the highest inhibition on BCSCs among the treatment. Given that HER2-directed therapy with Herceptin^®^ (trastuzumab) was effective to both HER2-positive and HER2-negative BCSCs [25], the addition of Herceptin to the liposomal formulation significantly enhanced the anticancer efficiency of LP-MET to BCSCs.

To test long-term cytotoxicity of various formulations, a clonogenic colony formation assay was carried out. Consistent with the result of MTT assay, Her-LP-MET effectively inhibited colony formation of BCSCs, and the combination therapy of Her-LP-MET and DOX inhibited the proliferation of BCSCs (Figure 5). The results of MTT assay and clonogenic colony formation assay indicate that Her-LP-MET showed the most effective inhibition of BCSCs among the tested treatments both in short-term and long-term.

A wound-healing assay was performed to determine migration of BCSCs. Inhibition of Her-LP-MET on BCSC migration was highest among the treatment of MET formulations (Figure 6). Furthermore, inhibition of BCSC migration was enhanced when combining Her-LP-MET with DOX. Since population cell migration is closely related to cancer invasion and metastasis, angiogenesis, immune responses, wound repair, and embryonic morphogenesis [39], the result of wound-healing assay indicate that co-treatment of Her-LP-MET and DOX is expected to not only kill BCSCs, but also its suppress cancer development.

Finally, an in vivo antitumor study was carried out to determine anticancer efficacy of various MET and DOX formulations. In contrast to the in vitro MTT study, LP-MET was shown have greater anti-cancer efficacy than MET (Figure 7). This is presumably because the PEGylated nano-liposomal system increases the stability and circulation time of MET in the blood, resulting in elevated bioavailability of MET. Furthermore, attachment of Herceptin on the surface of liposome might enhance in vivo anticancer effect of LP-MET. Interestingly, some of the xenograft mice treated with DOX alone developed metastasized tumors or died, whereas the combined treatment reduced the size of tumor and prevented recurrence more effectively than other treatments in a xenograft mouse model (Data not shown). However, this is not totally surprising as the maximum tolerated dose of free Dox in mice is ~6 mg/kg [40,41]. It is expected that antitumor efficacy and safety of the formulation could be improved if MET and DOX were incorporated together in liposome (2 drug-in-1 formulation) because the maximum tolerated dose of DOX when encapsulated into liposomes jumps up quite dramatically to ~55 mg/kg [42], and this needs further investigations.

## 5. Conclusions

Herceptin-conjugated liposomal MET delivery system could be considered as a promising strategy for novel anticancer formulation of MET. Additionally, combined treatment of DOX with Her-LP-MET could overcome limitations of current anti-BCSC therapies by specific targeting BCSCs.

## Figures and Tables

**Table 1:** Encapsulation efficiency (%), particle size (nm), and zeta potential (mV) of formulations.

Formulation	Her-LP	LP-MET	Her-LP-MET
Encapsulation efficiency (%)			21.6 ± 2
Particle size (nm)	52 ± 32	132 ± 49	159 ± 17
Zeta potential (mV)	−4.8 ± 4.2	−8.7 ± 1.3	−10.6 ± 7.6
